# Study on the promoting effect of microplastic exposure on prostate cancer migration and invasion based on network toxicology and single-cell sequencing technology

**DOI:** 10.3389/fonc.2026.1973812

**Published:** 2026-10-09

**Authors:** Haiyang Jiang, Ying Bi, Longguo Dai, Yong Zhu, Ruikang Zhang, Chen Hu, Hong Yang, Jun Li, Chongjian Zhang, Yu Bai, Ruiqian Li

**Affiliations:** 1 Department of Urology I, The Third Affiliated Hospital of Kunming Medical University(Peking University Cancer Hospital Yunnan, Yunnan Cancer Hospital, Cancer Center of Yunnan Province), Kunming, China; 2 School of Clinical Medicine, Haiyuan College of Kunming Medical University, Kunming, China

**Keywords:** acetyl tributyl citrate, cell invasion, cell migration, microplastic, prostate cancer

## Abstract

**Background:**

Prostate cancer (PCa) is a major malignant tumor threatening men’s global health. The plasticizer acetyl tributyl citrate (ATBC), widely used as a substitute for phthalates, is increasingly detected in human tissues; however, its role in PCa cell migration and invasion and the underlying mechanisms remain largely unknown.

**Methods:**

This study integrated network toxicology, scRNA-seq, transcriptomic analysis, Mendelian randomization (MR), and machine learning. *In vitro*, LNCaP cell proliferation, migration, and invasion were evaluated after ATBC treatment. ATBC toxicity targets were predicted using public databases. scRNA-seq data were analyzed to identify key cell clusters and candidate genes. Causal relationships between candidate genes and PCa were assessed by MR, and key genes were validated through multiple cohorts and HPA-based immunohistochemistry data. Molecular docking and virtual gene knockout were employed to explore binding affinity and functional impact.

**Results:**

In this study, *in-vitro* experiments were first performed, and it was found that the proliferation, migration and invasion abilities of ATBC-treated LNCaP cells were significantly altered. Subsequently, scRNA-seq data were analyzed to identify key cells (epithelial cells). Next, public databases were used to predict the toxic targets of ATBC, and the results of scRNA-seq and transcriptomic analyses were integrated to screen for potential target genes of ATBC in PCa. Finally, MR and machine-learning techniques were adopted to obtain the key genes (LPCAT1 and PDK1). In addition, molecular docking results for ATBC confirmed favorable binding affinity between the key genes and ATBC. Virtual knockout results also indicated that the key genes played a role in tumor migration and invasion.

**Conclusion:**

ATBC exposure may promote PCa migration and invasion by targeting LPCAT1 and PDK1, thereby perturbing cytoskeletal dynamics and metabolic pathways. These findings offer novel mechanistic insights into microplastic-associated PCa progression.

## 1 Introduction

Prostate cancer (PCa) is a malignant tumor that poses a severe threat to male health worldwide, having driven a sustained rise in male mortality and ranking second in incidence among all malignancies in men (1, 2). The etiology of PCa is multifaceted; established risk factors encompass advanced age, family history, inherited susceptibility genes, and hormonal imbalances. However, these factors do not fully account for the overall disease risk, underscoring the necessity to identify further contributing elements (3, 4). Accumulating evidence indicates that exposure to environmental pollutants, especially plastic contaminants increasingly detected in human tissues, plays a non-negligible role in the development and progression of PCa and various other cancers (5–7).

Plasticizers rank among the most vital additives in plastics and rubber, and these compounds are extensively utilized in consumer products and medical devices (8). Acetyl tributyl citrate (ATBC) is an organic compound synthesized via esterification from multiple plant-derived starting materials in the presence of catalysts. Owing to its low volatility, excellent cold resistance, and superior surface properties, ATBC has been widely adopted as an environmentally preferable substitute for toxic phthalate (PAE) plasticizers (8, 9). According to the toxicity evaluation by the European Food Safety Authority, which set a tolerable daily intake of 1.0 mg/kg body weight, ATBC is generally considered to have a high safety profile (10). Nonetheless, because ATBC is physically blended with polymers in plastics rather than chemically bonded, it readily migrates from food packaging, medical apparatus, and other plastic items, thereby contaminating the surrounding environment (11, 12). Studies have shown that microplastics can influence cell migration and invasion across diverse cancer types (13, 14). Moreover, microplastic polymers have been identified in human prostate tissue, covering tumor and paired noncancerous tissues of patients with prostate disorders, yet the inherent association between microplastics and PCa migration and invasion is still unclarified (15, 16).

Network toxicology, by harnessing powerful big data analytics within a bioinformatics framework, enables the systematic examination of toxicant effects on biological systems and the prediction of potential toxicity toward organisms, thus aiding the elucidation of toxicological mechanisms (17). Furthermore, integrating transcriptomics with single-cell analysis not only contributes to the identification of cancer biomarkers but also allows for the precise localization of environmental pollutant targets across distinct tumor cell populations, thereby deepening the understanding of pathogenic processes (18).

The present study aimed to predict the potential toxicity targets of ATBC using a network toxicology approach. By integrating multiple PCa transcriptomic datasets with single-cell sequencing data, differential expression analysis, machine learning, and Mendelian randomization-based causal inference were employed to systematically identify key genes associated with both ATBC exposure and PCa development. The functions of these genes and their expression profiles within malignant epithelial subpopulations were further explored. The findings of this study are expected to offer new scientific evidence regarding the regulatory mechanisms of microplastics in PCa and their impact on cell migration and invasion.

## 2 Materials and methods

### 2.1 Data sources

First, three PCa transcriptomic datasets were retrieved from the GEO (https://www.ncbi.nlm.nih.gov/geo/). Among them, GSE46602 (GPL570, 36 PCa vs. 14 controls) was employed as the training set, while GSE38241 (GPL4133, 18 PCa vs. 21 controls) and GSE316750 (GPL21290, 41 PCa vs. 41 controls) were designated as independent external validation sets. Subsequently, six single-cell RNA sequencing (scRNA-seq) datasets were obtained from the same database: GSE181294 (18 PCa vs. 21 controls), GSE193337 (4 PCa vs. 4 controls), GSE176031 (7 PCa vs. 8 controls), GSE153892 (3 PCa vs. 3 controls), GSE221603 (7 PCa vs. 3 controls), and GSE185344 (7 PCa vs. 7 controls). After integration, a total of 92 samples were collected, comprising 46 PCa samples and 46 control samples.

Next, PCa-related targets were searched in the GeneCards database (https://www.genecards.org/, relevance score > 8) (19) and the OMIM database (https://omim.org/) using “prostatic carcinoma” as the keyword. After merging and removing duplicates, 3,232 non-redundant PCa-related targets were obtained. In addition, GWAS summary data for PCa were acquired from the IEU Open GWAS database (https://gwas.mrcieu.ac.uk/) under the dataset ID ebi-a-GCST90018905, which included 11,599 PCa cases and 199,628 healthy controls (211,227 individuals in total), with 24,119,306 SNPs. The population was of European ancestry and the data were released in 2021.

### 2.2 Single-cell analysis

#### 2.2.1 Data preprocessing and quality control

The R package Seurat (v5.3.0) (20) was first applied for quality control and preprocessing of the dataset (**Supplementary Table 1**). Doublets were identified and removed using the R package scDblFinder (v1.22.0) (21). Subsequently, the HarmonyIntegration function from Seurat was utilized to perform batch correction for each dataset.

#### 2.2.2 Cell clustering and annotation

The integrated data were normalized with the LogNormalize method, and the top 2,000 highly variable genes (HVGs) were selected. Principal component analysis (PCA) was then carried out, and the optimal dimensionality was determined by inspecting the linear dimensionality reduction plot generated via the JackStrawPlot function. The Elbow diagram, cumulative explained variance ratio plot, principal component marginal gain plot, and second-order derivative plot were produced using scPCselect (v0.1.0) (https://github.com/xiaoqqjun/scPCselect), and the elbow point was identified. Cells were clustered using the FindNeighbors function and visualized by UMAP. Cell type annotation was conducted using marker genes retrieved from the CellMarker 2.0 database (http://117.50.127.228/CellMarker/CellMarkerSearch.jsp), through which each cell cluster was assigned to a specific cell type, and the proportional composition of each cell type was determined.

#### 2.2.3 Enrichment analysis and functional scoring of individual cell clusters

To investigate the main biological functions in which each cell type is involved, functional enrichment analysis was performed based on the single-cell expression data using the ReactomeGSA package (v1.22.0) (22). The top 15 terms exhibiting the largest differences in enrichment scores across cell types were visualized. Furthermore, enrichment scores (AUCell values) of ATBC targets in single cells were calculated using the AUCell package (v1.30.1).

#### 2.2.4 Copy number variation analysis of individual cell clusters

Copy-number variation (CNV) scores for each cell were inferred using the R package “fastCNV” (v1.1.4) (23), with T cells, B cells and NK cells as references.

#### 2.2.5 Identification and analysis of key subclusters

The expression data of the key cells (with control cells removed) were extracted for a second round of clustering. The same workflow was followed for re-dimensionality reduction and re-clustering, and annotation was performed based on marker genes provided in the literature (24). Subsequently, AUCell was used to score the expression of genes associated with the core signaling pathway related to cell migration and invasion (TGF-β, KEGG map04350) within each subcluster; the subcluster with the highest score was identified as the key cell cluster. Using the median TGF-β score of this key cluster as the cutoff, the cells were divided into high- and low-score groups. Differentially expressed genes between the two groups were identified with the FindMarkers function (*p <* 0.05 and |avg_log_2_FC| > 0.25) (25), yielding DEGs-sc, which were subsequently subjected to KEGG enrichment analysis.

#### 2.2.6 Cell-cell communication analysis

To characterize intercellular communication, ligand-receptor interaction analyses were performed using the CellChat package (v2.1.2) with CellChatDB.human as the reference, separately for PCa and control cells derived from the single-cell dataset. Ligand-receptor axes associated with the regulation of cell migration and invasion were selected and displayed in bubble plots.

#### 2.2.7 Pseudotime analysis

To examine the expression patterns of key genes in the single-cell data, pseudotime trajectory analysis was conducted on the key cell subclusters using the slingshot package (v2.16.0), and the cell differentiation trajectory was visualized on a UMAP plot.

### 2.3 Differential expression analysis

Differential expression analysis between the PCa group and the control group in the training set GSE46602 was performed using the R package limma (v3.64.3), applying a threshold of p.value < 0.05 and |log_2_FC| > 0.5. The resulting differentially expressed genes (DEGs) were visualized via a volcano plot generated with ggplot2 (v3.5.2).

### 2.4 Prediction of ATBC toxicity target genes

The chemical structure and SMILES notation of ATBC were obtained from the PubChem database (https://pubchem.ncbi.nlm.nih.gov/). The physicochemical properties and pharmacokinetic characteristics of ATBC were evaluated using the SwissADME database (http://www.swissadme.ch/). Potential targets of ATBC were predicted through the ChEMBL (https://www.ebi.ac.uk/chembl/), SwissTargetPrediction (http://www.swisstargetprediction.ch/, probability > 0), and STITCH (http://stitch.embl.de/, minimum required interaction score: 0.4) databases. The retrieved UniProt IDs were then converted to standard gene names via the UniProt database (https://www.uniprot.org/). After merging the targets from the three sources and removing duplicates, the potential ATBC toxicity targets were obtained.

### 2.5 Identification and analysis of candidate genes

First, the PCa-related targets, DEGs-sc, DEGs, and ATBC toxicity targets were intersected. The overlapping genes were designated as candidate genes and visualized using the UpSetR package. Next, to explore the biological functions and signaling pathways involving these candidate genes, Gene Ontology (GO) enrichment analysis and Kyoto Encyclopedia of Genes and Genomes (KEGG) pathway enrichment analysis were conducted using the R package clusterProfiler (v4.16.0) (*p <* 0.05) (26). Subsequently, the candidate genes were submitted to the STRING database (https://string-db.org/), and a protein-protein interaction (PPI) network was constructed with a threshold of interaction score > 0.15.

### 2.6 Mendelian randomization analysis

To examine the causal relationship between candidate genes and PCa, the harmonise_data function from the R package TwoSampleMR (v0.6.14) was used to align effect alleles and effect sizes, and the mr function was applied in conjunction with five complementary methods to conduct Mendelian randomization (MR) analysis. Expression quantitative trait locus (eQTL) data of the candidate genes served as the exposure, while PCa GWAS summary data (ebi-a-GCST90018905) were set as the outcome. The selection of instrumental variables (IVs) was guided by three core assumptions (1): IVs were strongly associated with the exposure (2); IVs were not directly associated with the outcome (3); IVs were independent of confounding factors. The screening criteria for IVs included a significance threshold of *p <* 5×10^–7^, removal of SNPs in linkage disequilibrium (LD) (r^2^ < 0.001, window size 100 kb), F-statistic > 10, and a minimum of three SNPs. Odds ratios (OR) were used to determine the direction of effect, with OR > 1 indicating a risk factor and OR < 1 indicating a protective factor. To evaluate the reliability of the MR results, sensitivity analyses were performed using TwoSampleMR, including heterogeneity testing (Cochran’s Q test), horizontal pleiotropy assessment (MR-Egger intercept test), and leave-one-out analysis. Finally, MR-Steiger directionality test was employed to confirm the causal direction, and only genes whose OR direction was consistent with the differential expression direction were retained for subsequent analyses.

### 2.7 Machine learning

To further identify key genes, the expression matrix of the causal candidate genes in the training set was subjected to feature selection through three distinct machine learning algorithms. First, LASSO regression was performed with the R package glmnet (v4.1.10) (27). Ten-fold cross-validation was adopted to determine the optimal regularization parameter λ, and genes whose coefficients were not shrunk to zero were retained as informative features. Second, SVM-RFE (5-fold cross-validation) was carried out using the caret package (v7.0.1) (https://doi.org/10.18637/jss.v028.i05) to obtain the feature subset that yielded the highest prediction accuracy. Third, random forest-based feature selection was implemented through the Boruta algorithm in the Boruta package (v9.0.0) (https://doi.org/10.18637/jss.v036.i11), retaining genes with significantly higher importance scores than shadow features. The genes intersecting across the three algorithmic outcomes were defined as candidate key genes.

### 2.8 Expression analysis of key genes

In the training set (GSE46602) and two independent external validation sets (GSE38241, GSE316750), the Wilcoxon rank-sum test from the rstatix package (v0.7.2) was applied to compare the expression differences of the candidate key genes between the PCa and control groups (*p <* 0.05). Genes that exhibited statistically significant differences with consistent expression trends were designated as key genes.

### 2.9 Human Protein Atlas analysis

To investigate the protein expression profiles of key genes across tissues and organs at both the RNA and protein levels, immunohistochemical staining images of proteins encoded by key genes in normal prostate tissue and prostate tumor tissue were retrieved from the Human Protein Atlas (HPA, https://www.proteinatlas.org/). These images were used to validate expression differences at the protein level.

### 2.10 GSEA enrichment analysis

To elucidate the biological functions and pathways in which key genes are involved in PCa, Spearman correlation coefficients between each key gene and all other genes were calculated using the psych package (v2.5.6) based on all samples in the training set, with the KEGG gene sets (https://www.gsea-msigdb.org/gsea/msigdb/) from the MSigDB database serving as the reference. The resulting coefficients were then ranked, and gene set enrichment analysis (GSEA) was conducted using clusterProfiler (|NES| > 1 and FDR < 0.05).

### 2.11 Immune microenvironment analysis

The single-sample gene set enrichment analysis (ssGSEA) algorithm implemented in the GSVA package (v2.2.0) was employed to quantify the infiltration abundance of 28 immune cell types in the training set samples (28). Differences in immune cell enrichment scores between the PCa group and the control group were subsequently compared using the Wilcoxon rank-sum test from the rstatix package (*p <* 0.05). The correlations between key gene expression levels and the infiltration scores of differentially enriched immune cells were then evaluated by Spearman correlation analysis via the psych package (|cor| > 0.3 and *p <* 0.05).

### 2.12 Molecular docking analysis

To determine the binding affinity between key genes and ATBC, the molecular structure of ATBC was downloaded from the PubChem database, and the three-dimensional structures of the proteins encoded by the key genes were obtained from the AlphaFold database (https://alphafold.com/). Molecular docking was performed using the CB-Dock2 online platform (https://cadd.labshare.cn/cb-dock2/php/index.php), with a binding energy lower than -5.0 kcal/mol considered indicative of favorable binding.

### 2.13 Virtual knockout analysis

To further infer the biological functions of target genes and their associated signaling pathways, virtual knockout of the key genes was conducted with the scTenifoldKnk package (v1.0.2). Genes that displayed significant expression perturbations after virtual knockout were identified (*p <* 0.05). Subsequently, GO and KEGG enrichment analyses were performed on these significantly perturbed genes to deduce the biological functions of the key genes and the pathways in which they are involved (*p <* 0.05).

### 2.14 Experimental methods

#### 2.14.1 Cell culture

Human prostate cancer cells (LNCaP) were cultured in RPMI-1640 basal medium (PM150110, Promosys, China Wuhan) supplemented with 10% fetal bovine serum (FBS, 10091-148, Gibco, China), 100 U/mL penicillin, and 100 µg/mL streptomycin at 37 °C with 5% CO_2_.

#### 2.14.2 CCK-8 assay

Groups included blank control, 0.1% DMSO vehicle, and different concentration (1 nM, 10 nM, 100 nM, 1,000 nM and 10,000 nM) ATBC treatments (Sigma, Shanghai, China). After 48 h in phenol red-free medium, 10 μL CCK-8 reagent (Proteintech, Wuhan, China) was added per well, and absorbance at 450 nm was measured when the color stabilized.

#### 2.14.3 Cell scratch assay

LNCaP cells were seeded in 6-well plates. A sterile 200 μL pipette tip was used to vertically scratch the cell monolayer. The detached cells were then washed away with PBS, and ATBC at different concentrations was subsequently added according to each group. Wound images were captured under a microscope at 0, 6, 12, 24, and 48 hours of incubation.

#### 2.14.4 Cell migration assay

LNCaP cells in logarithmic growth phase were resuspended in serum-free medium, and the cell suspension was added to the upper Transwell chamber. The lower chamber was filled with complete medium containing ATBC (Sigma, Shanghai, China). After 48 hours of incubation, the migrated cells were fixed, stained with a 0.1% crystal violet solution (C8470, Solarbio, Beijing, China), and quantified using ImageJ software.

#### 2.14.5 Invasion experiment

Transwell chambers were coated with 1 mg/mL of Matrigel (G4131, Servicebio, Wuhan, China) and incubated at 37 °C for 1 h. After hydration, LNCaP cells (5 × 10^3^ cells) suspended in serum-free medium were mixed with the sample working solution and added to the upper chamber. Complete medium containing different concentrations of ATBC was loaded into the lower chamber. After 48 h of incubation, the invaded cells were fixed, stained with 0.1% crystal violet (C8470, Solarbio, Beijing, China), and photographed under a microscope. The number of invaded cells was quantified using ImageJ software.

### 2.15 Statistical analysis

All statistical analyses were conducted using R software (v4.3.0) and GraphPad Prism (v10.1.2). Each experiment was repeated independently no fewer than three times, and data are expressed as the mean ± standard deviation (SD). The Wilcoxon rank-sum test was applied for comparisons between two groups, and Spearman’s rank correlation coefficient was employed for correlation analyses. A p-value of less than 0.05 was regarded as statistically significant.

## 3 Results

### 3.1 Effects of ATBC on prostate cancer LNCaP cells

To investigate the impact of the environmental pollutant ATBC on PCa cells, the proliferation, migration, and invasion abilities of human prostate cancer LNCaP cells treated with ATBC were assessed. The CCK-8 assay results first showed that ATBC at concentrations of 100 nM and 1000 nM significantly promoted LNCaP cell proliferation compared with the blank control group (*p <* 0.05) (**Figure 1A**). Subsequently, the Cell scratch assay demonstrated that, at 48 hours, wound closure in both the 100 nM and 1000 nM ATBC treatment groups was significantly greater than that in the control group (*p <* 0.05) (**Figure 1B**). Consistently, the migration assay revealed that the number of migrated cells in the 100 nM and 1000 nM ATBC groups was significantly higher than that in the control group after treatment (*p <* 0.05) (**Figure 1C**). Furthermore, the cell invasion assay showed that the number of invaded cells was significantly increased in both the 100 nM and 1000 nM ATBC treatment groups (*p <* 0.05) (**Figure 1D**). These findings indicate that ATBC can enhance the migratory and invasive capabilities of LNCaP cells and may contribute to PCa progression by promoting malignant cell behaviors, suggesting its potential role as an environmental pollutant in the advancement of PCa.

**Figure 1:**
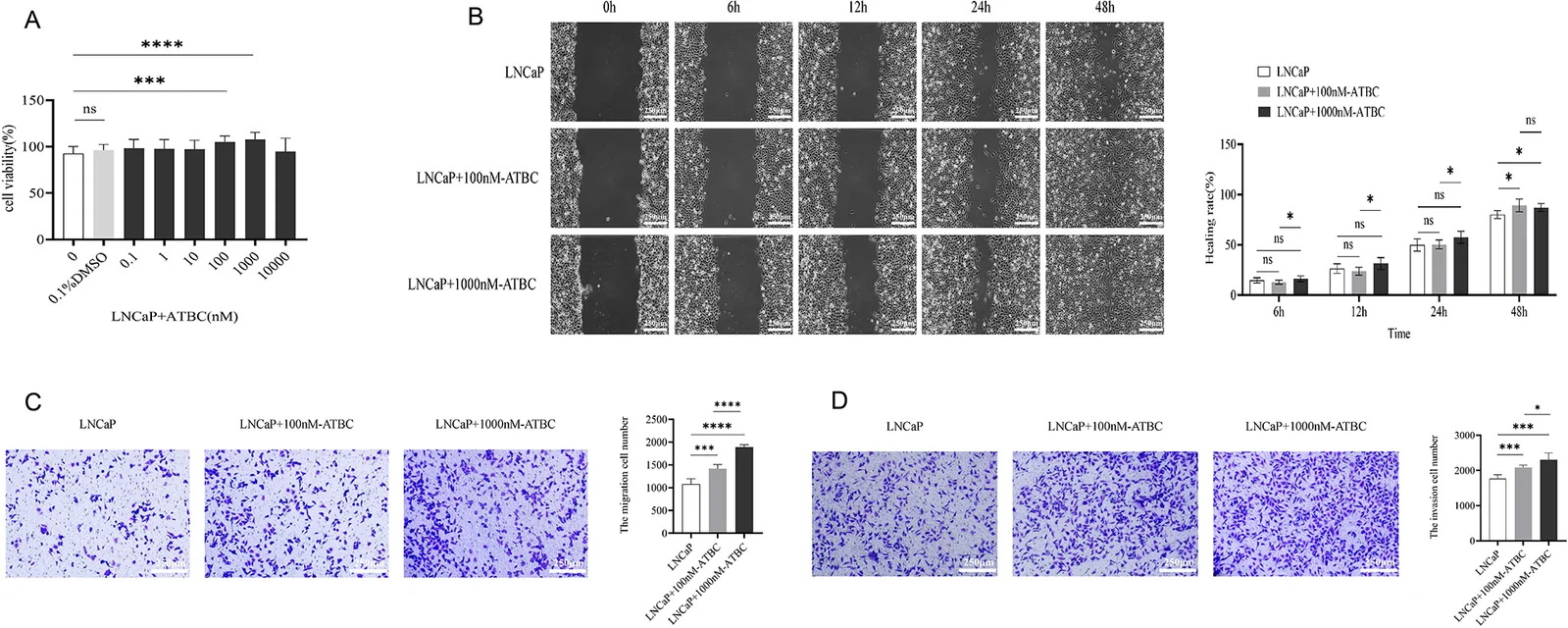
Effects of Acetyl tributyl citrate (ATBC) treatment on LNCaP cells. **(A)** Histogram of CCK-8 assay results, ns represents insignificant, *p<0.05, **p<0.01, ***p<0.001, ****p<0.0001. **(B)** Photographs and histogram of wound-healing assay results, ns represents insignificant, *p<0.05. **(C)** Images and histogram of cell migration assay results, ns represents insignificant, *p<0.05, **p<0.01, ***p<0.001, ****p<0.0001. **(D)** Images and histogram of cell invasion assay results, ns represents insignificant, *p<0.05, **p<0.01, ***p<0.001.

### 3.2 Single-cell analysis and identification of key cells

To explore gene expression profiles and key cell types in PCa at the single-cell level, quality control was first performed on the single-cell dataset, yielding 271,005 high-quality cells (**Supplementary Figure 1**, **Supplementary Tables 2, 3**). Based on the top 2,000 highly variable genes, the optimal dimensionality (30 principal components) was determined by integrating the linear dimensionality reduction plot and the scree plot, and cell clustering was conducted at a resolution of 0.5 (**Figure 2A**; **Supplementary Figure 2**). The clustering analysis identified 20 cell subclusters, which were annotated into 11 cell types according to marker genes (**Figures 2B, C**, **Table 1**). Cell proportion analysis revealed that T cells accounted for the largest fraction, whereas epithelial cells exhibited the greatest proportional increase in the PCa group relative to the control group (**Figure 2D**), suggesting a potentially important role in disease progression. Enrichment analysis across different cell types showed that epithelial cells and macrophages were highly enriched in multiple pathways (**Figure 2E**). The AUCell scores demonstrated that ATBC targets exhibited relatively high scores in epithelial cells, macrophages, and fibroblasts (**Figure 2F**). CNV analysis indicated that the CNV scores of epithelial cells were significantly higher than those of other cell types (**Figures 2G, H**). Based on these integrated findings, epithelial cells were identified as the key cell type for subsequent analyses.

**Figure 2:**
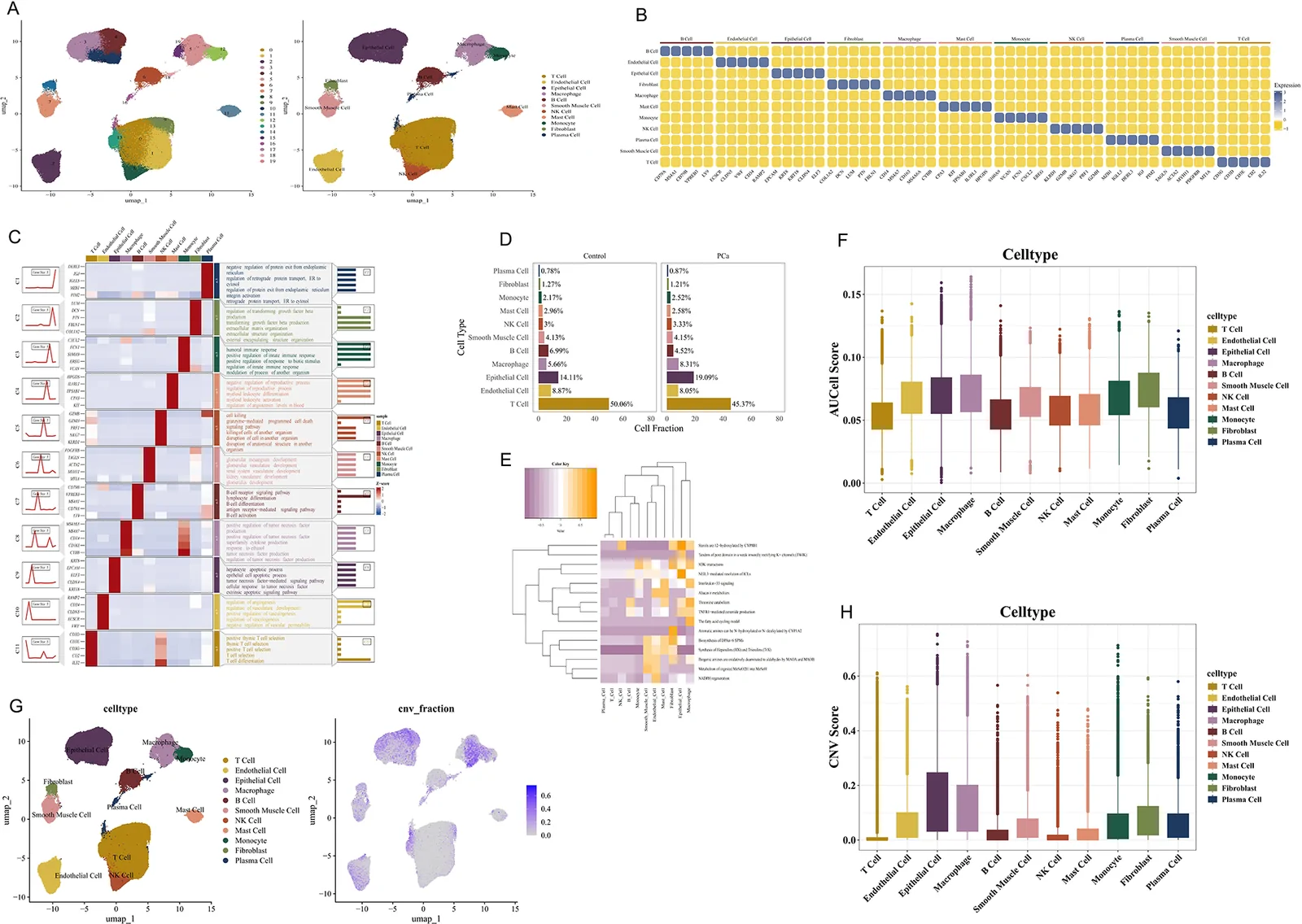
Screening and identification of key cells. **(A)** UMAP clustering plots before and after cell annotation, with different colors corresponding to different cell clusters. **(B)** UMAP plot after cell-type annotation using marker genes. **(C)** Heatmap showing the expression of characteristic marker genes for each cell type, with red indicating high expression and purple indicating low expression. **(D)** Bar chart illustrating the proportions of each cell type in the control group and PCa group. **(E)** Heatmap for enrichment analysis of different cell types. **(F)** Box plot presenting the Copy Number Variation (CNV) scores of different cell populations. **(G)** Paired UMAP visualization of cell annotation results and corresponding CNV scores. **(H)** Distribution of CNV scores across different cell types.

**Table 1:** Single-cell annotation marker genes.

Cell	Marker	Clusters
Plasma Cell	MZB1, IGLL5, DERL3, IGJ, PIM2	16,18
Fibroblast	COL1A2, DCN, LUM, PTN, FBLN1	14
Monocyte	S100A9, VCAN, FCN1, CXCL2, EREG	12
Mast Cell	CPA3, KIT, TPSAB1, IL1RL1, HPGDS	11
NK Cell	KLRD1, GZMB, NKG7, PRF1, GZMH	8
Smooth Muscle Cell	TAGLN, ACTA2, MYH11, PDGFRB, MT1A	7
B Cell	CD79A, MS4A1, CD79B, VPREB3, LY9	6
Macrophage	CD14, MS4A7, CD163, MS4A6A, CYBB	5,15,19
Epithelial Cell	EPCAM, KRT8, KRT18, CLDN4, ELF3	3,4,10,17
Endothelial Cell	ECSCR, CLDN5, VWF, CD34, RAMP2	2
T Cell	CD3G, CD3D, CD3E, CD2, IL32	0,1,9,13

### 3.3 Identification and analysis of key epithelial cell subclusters

Epithelial cells were subjected to secondary clustering analysis. After batch correction, the optimal dimensionality was determined by integrating the linear dimensionality reduction plot and the scree plot, and cell clustering was subsequently performed (**Supplementary Figure 3**). Four subclusters were identified and annotated into three cell types according to marker genes (**Figures 3A–C**, **Table 2**). The AUCell scores based on the TGF-β signaling pathway gene set then showed that Double-Negative Basal-like Epithelial Cells exhibited the highest score and were therefore designated as the key cell cluster (**Figure 3D**). Differential analysis between the high and low TGF-β scoring groups within this cluster yielded 5,717 differentially expressed genes (DEGs-sc). Subsequent KEGG enrichment analysis of the DEGs-sc revealed significant enrichment in pathways such as PI3K-Akt, MAPK, and TGF-β (**Figures 3E, F**).

**Figure 3:**
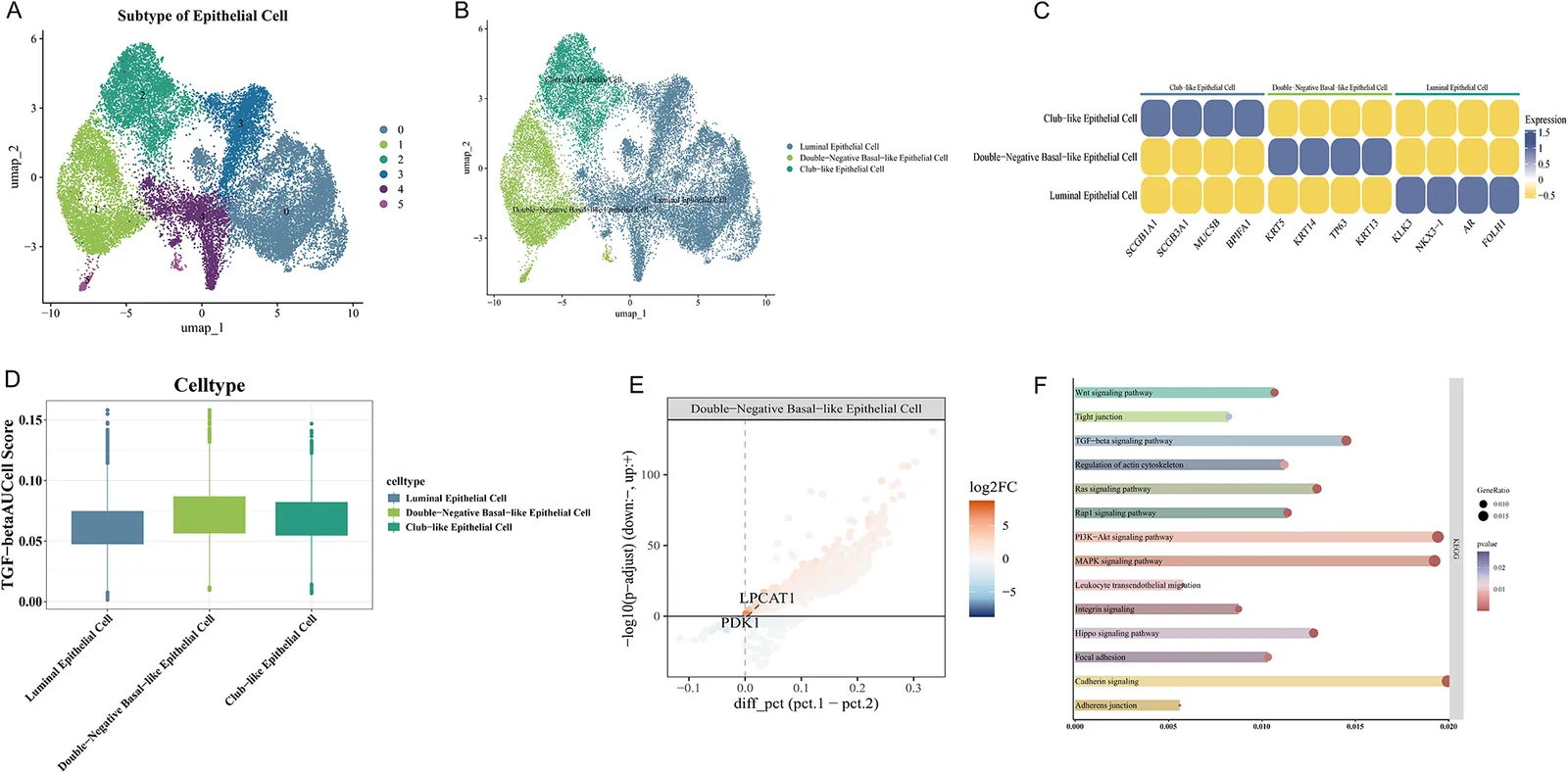
Clustering and analysis of key cell subpopulations. **(A, B)** UMAP plots before and after annotation of key cell subpopulations, with different colors representing distinct cell subpopulations; **(A)** before annotation; **(B)** after annotation. **(C)** Heatmap showing the expression of marker genes across different subpopulations, where blue indicates high expression and yellow indicates low expression. **(D)** TGF-beta level scores of different key cell subpopulations. **(E)** Differential analysis plot for groups with high and low TGF-beta expression in different key cell subpopulations. **(F)** Pathway enrichment score plot for different key cell subpopulations.

**Table 2:** Epithelial cell annotation marker genes.

Epithelial cell type	Marker genes
Luminal Epithelial Cell	The main secretory epithelial cells of the prostate gland express markers such as AR, NKX3-1, KLK3 and FOLH1
Club-like Epithelial Cell	It is named for its similar expression characteristics of secretoglobins (SCGB1A1, SCGB3A1, MUC5B, BPIFA1) to pulmonary Club cells. It is mainly distributed in the prostatic urethra and proximal ducts, and possesses functions of stress adaptation and immune regulation.
Double-Negative Basal-like Epithelial Cell	"Double-negative" refers to the absence of androgen receptor (AR) signaling and the expression of neuroendocrine (NE) markers. Although it exhibits a basal-like phenotype (expressing KRT5, KRT14, TP63 and KRT13), it actually represents a state of lineage plasticity originating from the luminal compartment.

### 3.4 Screening and enrichment analysis of candidate genes

Differential expression analysis was first performed on the training set, identifying 3,045 DEGs between PCa and normal tissues (**Figure 4A**; **Supplementary Table 4**). The physicochemical and drug-likeness properties related to ATBC toxicity were then characterized: ATBC (C_20_H_34_O_8_, molecular weight 402.48 g/mol) displayed good lipophilicity (Log P ≈ 3.29), and its aqueous solubility was predicted to be moderate (Log S -3.15 to -5.17). Gastrointestinal absorption was predicted to be high, whereas penetration across the blood-brain barrier was limited. Drug-likeness assessment indicated compliance with Lipinski’s and Ghose’s rules, while violations of Veber’s and Muegge’s rules were noted due to an excessive number of rotatable bonds; the bioavailability score was 0.55 (**Supplementary Figure 4**). By integrating prediction results from ChEMBL, SwissTargetPrediction, and STITCH, a total of 142 potential ATBC toxicity targets were obtained (**Supplementary Table 5**), and a compound-target network was constructed to illustrate the direct associations between ATBC and multiple target genes (**Figure 4B**). Finally, the intersection of DEGs-sc, DEGs, ATBC toxicity targets, and PCa-related targets yielded nine overlapping genes, which were designated as candidate genes (**Figure 4C**).

**Figure 4:**
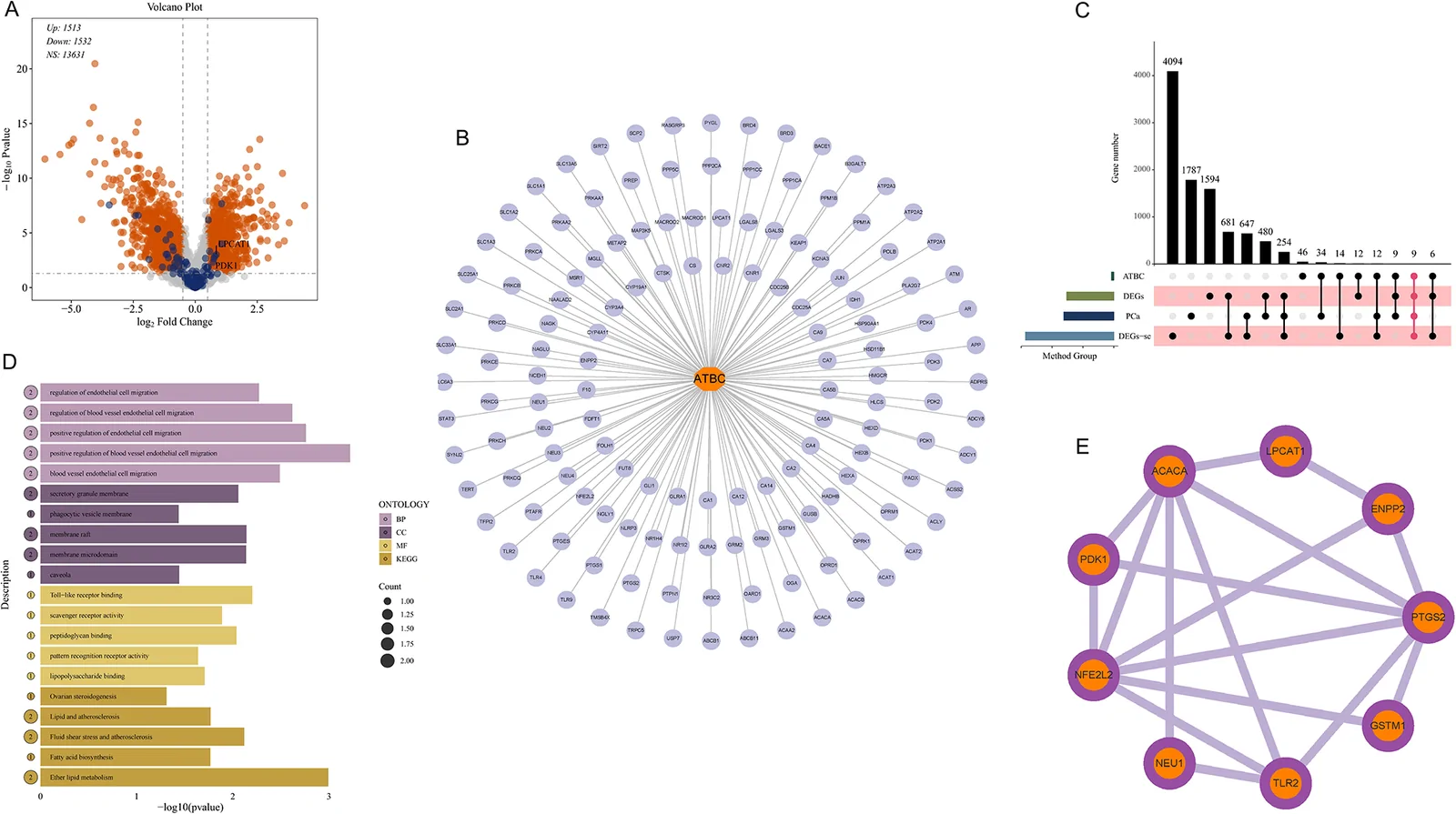
Screening and enrichment analysis of candidate genes. **(A)** Volcano plot for differential analysis between disease and control groups in the training set. **(B)** Network diagram of ATBC-action targets, with purple spheres representing target genes. **(C)** UpSet plot for obtaining candidate genes by intersection analysis. **(D)** Plot of enrichment analysis results for candidate genes. BP, Biological Process; CC, Cellular Component; MF, Molecular Function. **(E)** PPI network, where each node represents a gene and edges indicate interactions at the protein level.

Enrichment analysis was subsequently conducted for these nine candidate genes. GO analysis revealed significant enrichment in terms associated with cell migration and invasion, such as regulation of vascular endothelial cell migration, membrane raft, and Toll-like receptor binding. Consistently, KEGG analysis also showed significant enrichment in pathways related to cell migration and invasion, including ether lipid metabolism and lipid and atherosclerosis (**Figure 4D**; **Supplementary Tables 6**, **7**). PPI network analysis further demonstrated close interactions among the nine candidate genes (**Figure 4E**).

### 3.5 Mendelian randomization analysis

To investigate the causal relationship between candidate genes and PCa, bidirectional MR analysis was performed on the nine candidate genes. With the IVW method serving as the primary analysis and *p <* 0.05 as the significance threshold, three genes (LPCAT1, PDK1, and NEU1) were found to have significant causal associations with PCa after instrumental variable screening, all acting as risk factors (OR > 1) (**Figure 5A**). **Supplementary Table 8**–**10** summarized the F-statistics of instrumental SNPs for LPCAT1, PDK1, and NEU1 (F > 10). Scatter plots, forest plots, and funnel plots further corroborated these causal relationships (**Figures 5B–D**; **Supplementary Figures 5A–C**). Sensitivity analyses were subsequently conducted. Heterogeneity was observed for some genes (**Table 3**) and was addressed by adopting the IVW random-effects model. The MR-Egger intercept test indicated no significant horizontal pleiotropy (p > 0.05) (**Table 4**), and leave-one-out analysis confirmed the robustness of the results (**Figure 5E**; **Supplementary Figure 5D**). The MR-Steiger directionality test verified the correct causal direction between the candidate genes and PCa (**Table 5**). Finally, by aligning the OR direction with the differential expression direction, LPCAT1 and PDK1 (OR > 1 and logFC > 0) were retained for subsequent analysis.

**Figure 5:**
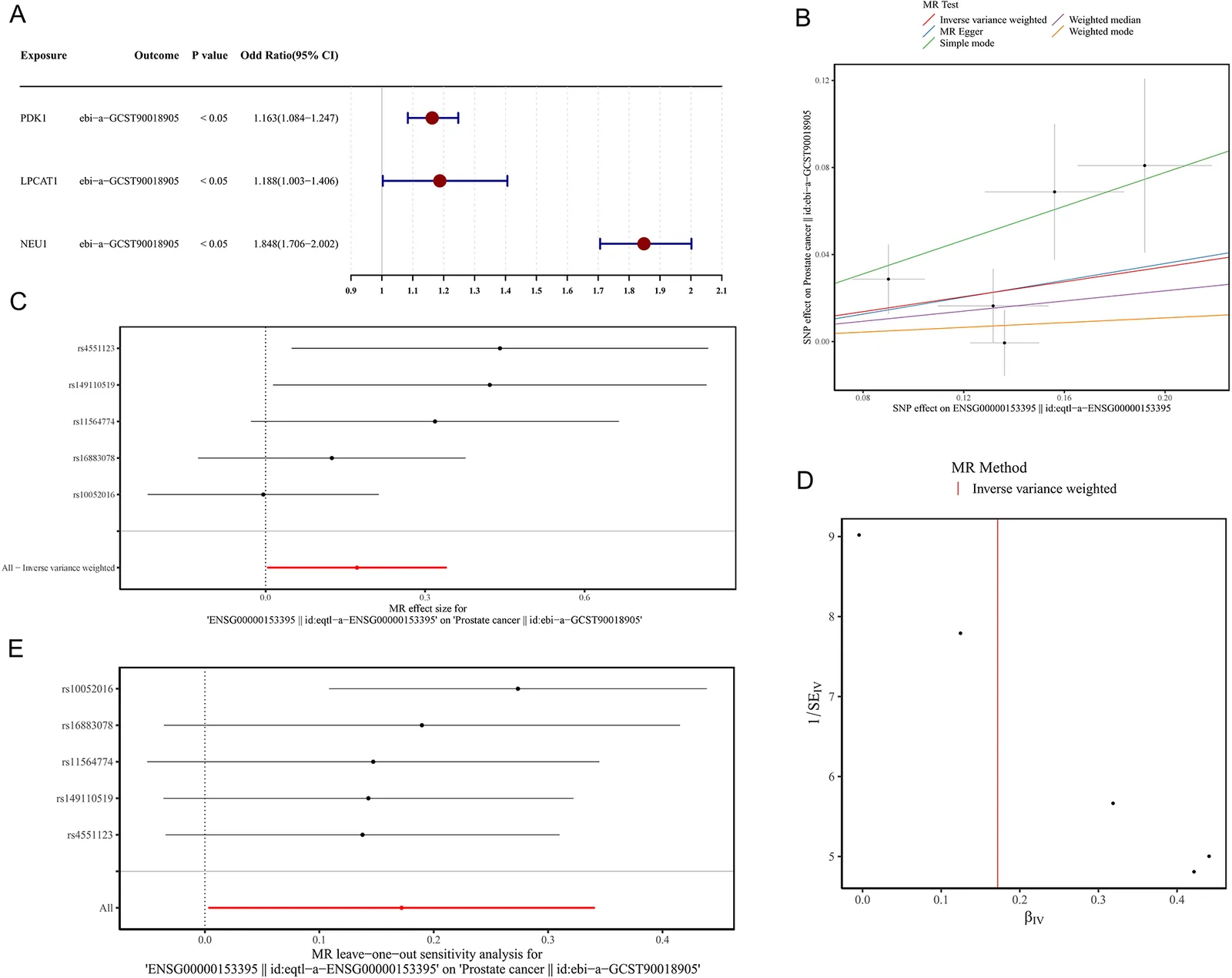
Mendelian randomization (MR) analysis of candidate genes. **(A)** Forest plot for MR analysis of candidate genes. CI, Confidence Interval. **(B–E)** MR scatter plot **(B)**, forest plot **(C)**, funnel plot **(D)**, and leave-one-out plot **(E)** for genes LPCAT1.

**Table 3:** Results of the mendelian randomization (MR) heterogeneity test analysis.

id.exposure	id.outcome	Q	Q_df	Q_pval
LPCAT1	Prostate cancer || id:ebi-a-GCST90018905	6.60704471	4	0.15816942
PDK1	Prostate cancer || id:ebi-a-GCST90018905	30.3582907	9	0.00038119
NEU1	Prostate cancer || id:ebi-a-GCST90018905	11.0311317	12	0.52625259

**Table 4:** Results of the mendelian randomization (MR) horizontal pleiotropy test analysis.

id.exposure	id.outcome	egger_intercept	se	pval
LPCAT1	Prostate cancer || id:ebi-a-GCST90018905	-0.00287562	0.06233367	0.96610369
PDK1	Prostate cancer || id:ebi-a-GCST90018905	0.064574192	0.03253908	0.08247078
NEU1	Prostate cancer || id:ebi-a-GCST90018905	-0.04945837	0.05485422	0.38656807

**Table 5:** Results of the Steiger’s directional test.

id.exposure	id.outcome	correct_causal_direction	steiger_pval
LPCAT1	Prostate cancer || id:ebi-a-GCST90018905	TRUE	6.32E-44
PDK1	Prostate cancer || id:ebi-a-GCST90018905	TRUE	0
NEU1	Prostate cancer || id:ebi-a-GCST90018905	TRUE	8.9383744E-173

### 3.6 Identification and validation of key genes

Based on the expression matrices of LPCAT1 and PDK1 in the training set, three machine learning algorithms were employed for further feature selection. LASSO regression determined the optimal λ value (λ = 0.0313) through ten-fold cross-validation and retained both LPCAT1 and PDK1 (**Figures 6A, B**). SVM-RFE analysis showed that the highest model accuracy (Accuracy = 0.799) was achieved when two features were selected, yielding LPCAT1 and PDK1 (**Figure 6C**). The Boruta algorithm confirmed that the importance scores of LPCAT1 and PDK1 were significantly higher than those of shadow features (**Figure 6D**). The intersection of the results from the three algorithms was then extracted, and the overlapping genes, LPCAT1 and PDK1, were identified as candidate key genes (**Figure 6E**).

**Figure 6:**
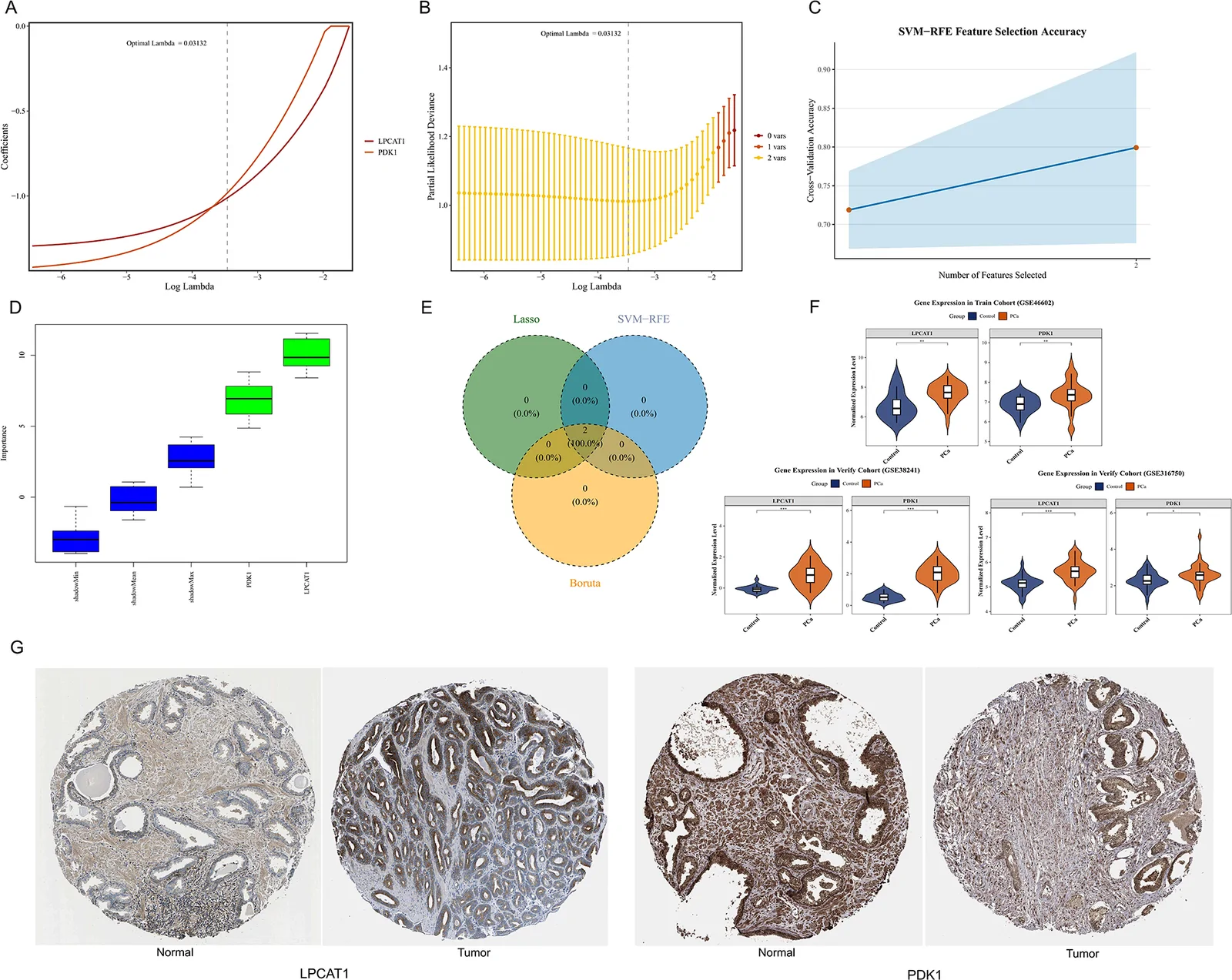
Machine-learning-based identification and expression validation of key genes. **(A)** Least absolute shrinkage and selection operator (LASSO) regression coefficient screening plot. **(B)** Lasso regression cross-validation plot. **(C)** Accuracy curve of feature screening by the Support vector machine-recursive feature elimination (SVM-RFE) algorithm. **(D)** Box plot of importance scores for all candidate genes evaluated by the Boruta algorithm. **(E)** Venn diagram showing the intersection of screening results from the LASSO, SVM-RFE and Boruta algorithms. **(F)** Box plot for expression differences of candidate key genes between the Prostate cancer (PCa) group and normal control group in the training and validation sets, ns represents insignificant, *p<0.05, **p<0.01, ***p<0.001. **(G)** Expression-level plot of key genes in PCa tissues versus control tissues from the HPA database.

Subsequently, the expression differences of these genes between the PCa group and the control group were examined in the training set and independent external validation sets. The results showed that both LPCAT1 and PDK1 exhibited statistically significant differences with consistent expression trends across datasets, and they were therefore designated as key genes (**Figure 6F**). In addition, immunohistochemical staining data from the HPA database confirmed a marked upregulation of LPCAT1 at the protein level in tumor tissues, whereas no significant change was observed in PDK1 staining intensity (**Figure 6G**).

### 3.7 GSEA enrichment analysis and immune microenvironment analysis of key genes

To systematically dissect the mechanisms through which LPCAT1 and PDK1 regulate tumor migration and invasion, GSEA was performed separately for each gene. The results revealed that both genes were enriched in the ubiquitin-mediated proteolysis pathway, which can indirectly modulate cell migration (**Figure 7A**). Moreover, LPCAT1 exhibited a pronounced upregulation in pathways highly relevant to cell migration and invasion, such as regulation of the actin cytoskeleton and ECM-receptor interaction, and it also positively regulated cancer-related pathways. PDK1 was enriched in the calcium signaling pathway associated with cell migration signal transduction, while significantly downregulating oxidative phosphorylation and the TGF-β signaling pathway (**Figure 7A**; **Supplementary Tables 11**, **12**).

**Figure 7:**
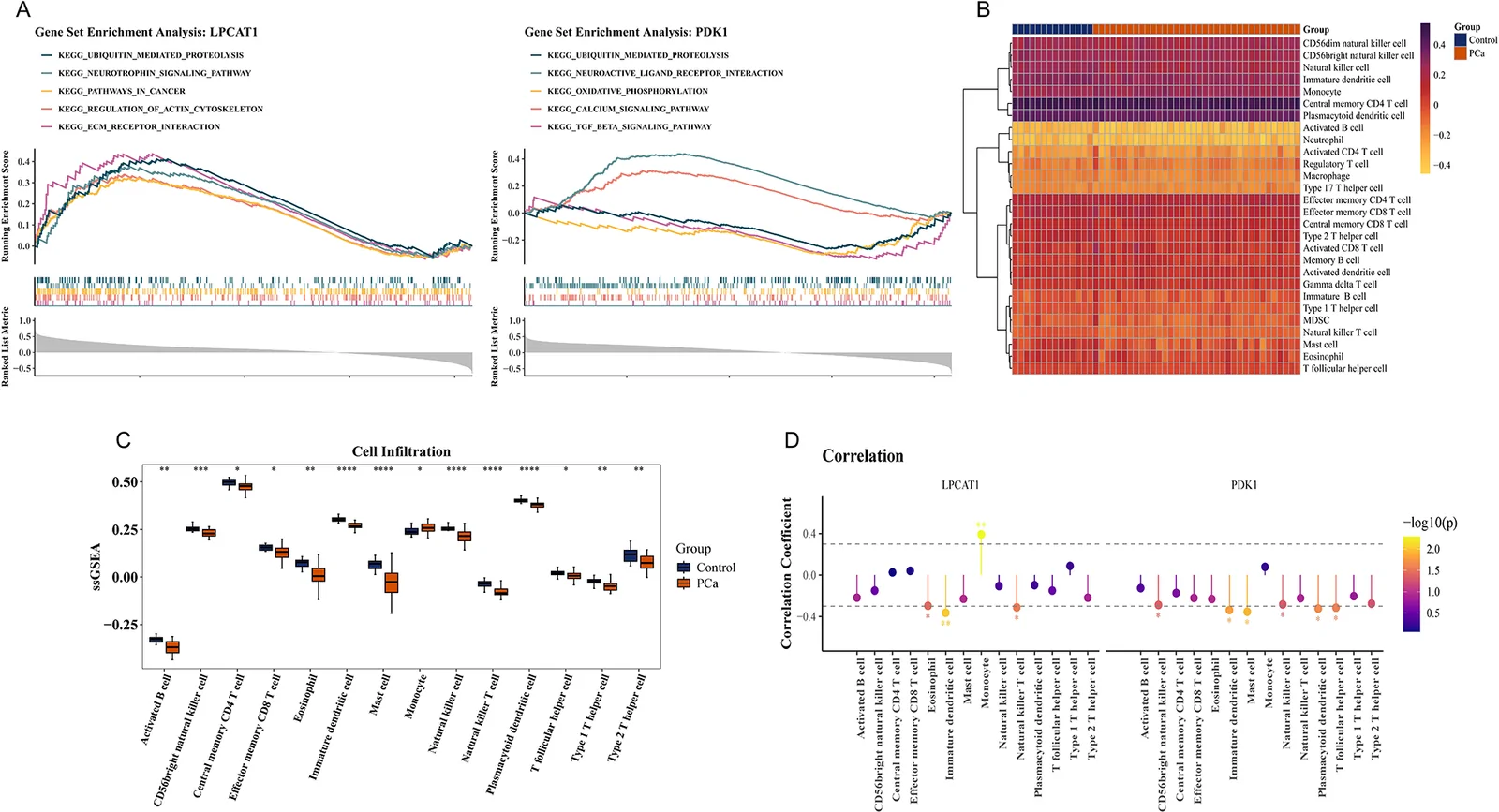
Gene set enrichment analysis (GSEA) broad inst. enrichment analysis and immune microenvironment analysis of key genes. **(A)** GSEA enrichment analysis of genes LPCAT1 and PDK1. This figure has three parts. Part 1: Five top broken lines form the gene Enrichment Score line graph. Its vertical axis shows Running ES; the graph’s peak gives the gene set’s Enrichment score, with pre-peak genes as core genes. The horizontal axis marks each gene in the set, matching Part 2’s barcode-style vertical lines. Part 2: The barcode-like Hits section, where each vertical line stands for one gene in the set. Part 3: Rank-value distribution diagram for all genes; its vertical axis is the gene-sorting ranked list metric. **(B)** Heatmap of enrichment scores for immune-infiltrating cells. Blue represents control group samples and red represents PCa group samples. **(C)** Box plot of differential immune cells, *p<0.05, **p<0.01, ***p<0.001, ****p<0.0001. **(D)** Lollipop plot showing the correlation between key genes and differential immune cells, *p<0.05, **p<0.01, ***p<0.001, ****p<0.0001.

Next, the immune microenvironment of PCa was analyzed. The results showed significant differences in the enrichment scores of 14 immune cell types between the PCa group and the control group (*p <* 0.05) (**Figures 7B, C**). Spearman correlation analysis indicated that LPCAT1 exhibited the strongest positive correlation with monocytes (cor = 0.39, *p =* 0.0051) and the strongest negative correlation with immature dendritic cells (cor = -0.36, *p =* 0.0098). PDK1 displayed the strongest negative correlation with mast cells (cor = -0.35, *p =* 0.0118) (**Figure 7D**; **Supplementary Table 13**), suggesting that key genes may influence PCa progression by modulating immune cell infiltration.

### 3.8 Molecular docking between key genes and ATBC

To evaluate the binding affinity between ATBC and the proteins encoded by the key genes, molecular docking was conducted. The results demonstrated that the binding energy of ATBC with LPCAT1 protein (AF-Q8NF37-F1) was -6.1 kcal/mol, and with PDK1 protein (AF-Q15118-F1) was -6.2 kcal/mol (**Figures 8A, B**), both lower than -5.0 kcal/mol. These findings indicate that ATBC possesses favorable binding activity toward both proteins.

**Figure 8:**
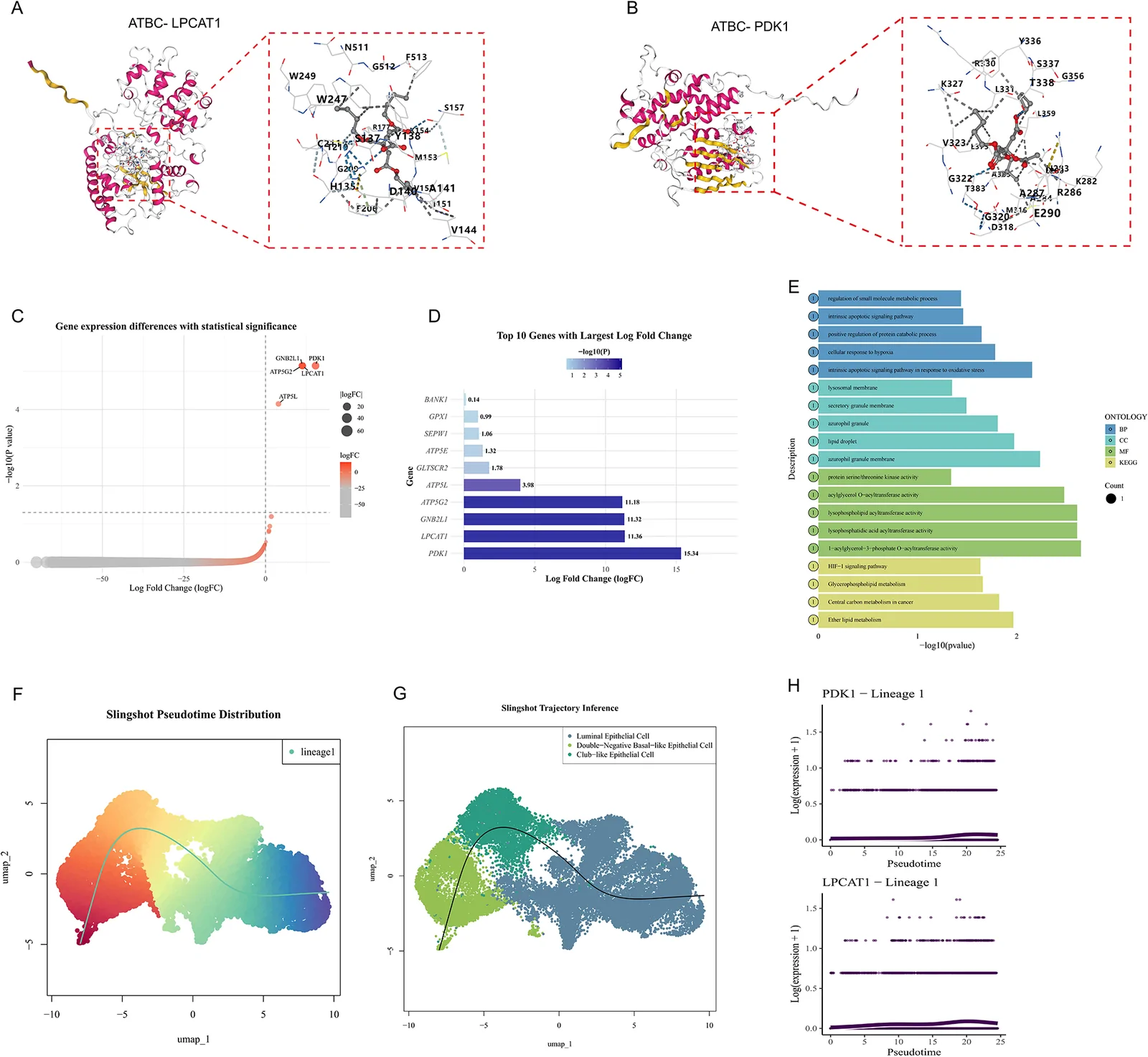
Molecular docking, virtual knockout and pseudotime-series analysis of key genes. **(A, B)** Molecular docking diagrams of gene LPCAT1 **(A)** and PDK1 **(B)**. **(C)** Bubble plot showing differential perturbations following LPCAT1 and PDK1 virtual knockout. **(D)** Bar chart of the top 10 genes with the highest upregulation levels after virtual knockout. **(E)** Functional enrichment plot of genes perturbed by knockout. BP, Biological Process; CC, Cellular Component; MF, Molecular Function. **(F)** Lineages derived from pseudotime analysis of epithelial cells. **(G)** Pseudotime differentiation trajectories of distinct epithelial cells. **(H)** Dynamic expression curves of key genes along the trajectories.

### 3.9 Virtual knockout analysis of key genes

Virtual knockout was performed for LPCAT1 and PDK1 individually, and a total of five significantly perturbed genes were identified (**Figures 8C, D**). GO and KEGG enrichment analyses revealed that these perturbed genes were enriched in signaling pathways related to metabolism, migration, and invasion (**Figure 8E**; **Supplementary Tables 14, 15**), further illustrating the driving roles of LPCAT1 and PDK1 in metabolic reprogramming and the progression of tumor migration and invasion in PCa.

### 3.10 Pseudotime dynamic expression analysis of key genes

Pseudotime trajectory inference was carried out on the key epithelial cell subclusters. The results showed that cells progressed from a red-coded state at low pseudotime, extending outward along a smooth curve, and gradually transitioned to a high-pseudotime state at the branch termini (**Figure 8F**). Integrated analysis of the trajectory and cell subclusters revealed that Double-Negative Basal-like Epithelial Cells were distributed at the early pseudotime stage, Club-like Epithelial Cells at the intermediate stage, and Luminal Epithelial Cells at the terminally differentiated state (**Figure 8G**). The key genes LPCAT1 and PDK1 maintained low baseline expression levels at the early differentiation stage, and as pseudotime progressed to the mid-to-late stage, their expression levels synchronously and gradually increased, reaching a peak in Luminal Epithelial Cells. This pattern suggests that the key genes may exhibit coordinated expression characteristics during epithelial cell differentiation (**Figure 8H**).

### 3.11 Communication analysis of key cells

Cell-cell communication analysis showed that intercellular communication activities were pronounced in both the PCa group and the control group. Among the cell types, T cells displayed the largest node, suggesting a potential role as a central communication hub. Dense bidirectional communication circuits were observed between epithelial cell subclusters and other cell populations (**Figures 9A, B**). The heatmap of communication counts revealed that high-frequency interactions between tumor epithelial subclusters and fibroblasts, macrophages, and monocytes were more prevalent in the PCa group, and the overall interaction abundance was slightly higher than that in the control group (**Figures 9C, D**). In addition, the ligand-receptor communication map indicated that, compared with normal tissues, PCa tissues exhibited more complex intercellular communication patterns, involving vascular remodeling, inflammatory responses, and migration/invasion (**Supplementary Figure 6**). Epithelial cells mainly emitted strong signals to endothelial cells, stromal cells, and other targets via VEGF, PDGFA, and GDF15, modulating biological processes such as angiogenesis, stromal remodeling, and cell migration. Conversely, immune cells such as macrophages and mast cells acted back on tumor epithelial cells through molecules like EREG and AREG, thereby forming paracrine loops that facilitate tumorigenesis (**Figures 9E, F**).

**Figure 9:**
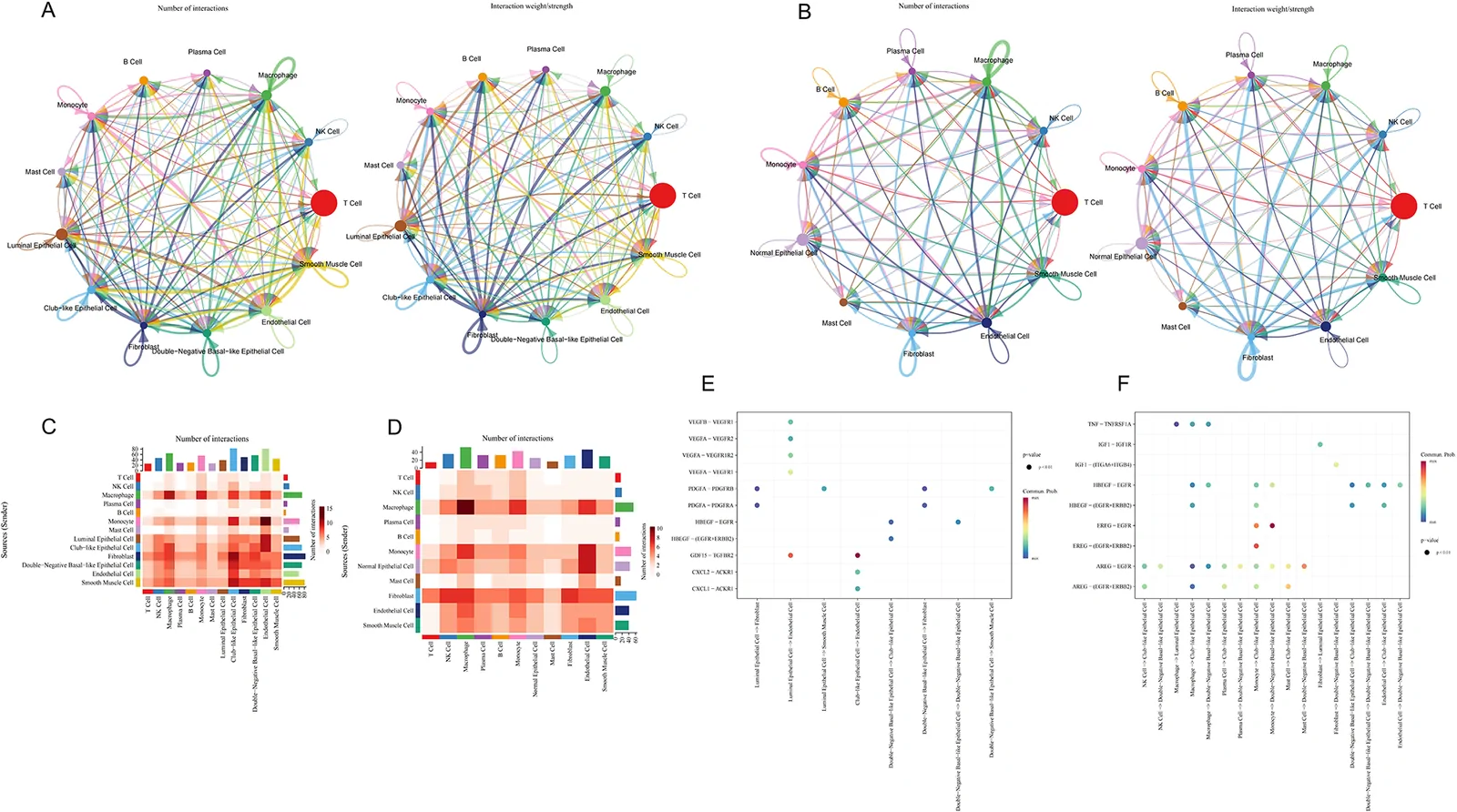
Cell-cell communication analysis. **(A)** Network diagram showing the number and weight of cell-cell interactions in the PCa group and the control group, where node size represents the total number or strength of communication events each cell participates in. **(B)** Network diagram showing the number and weight of cell-cell interactions in the control group, where node size represents the total number or strength of communication events each cell participates in. **(C, D)** Heatmaps of pairwise cell-cell interaction counts for the PCa group **(C)** and the control group **(D)**, with dark regions corresponding to higher numbers of interactions. **(E, F)** Bubble plots of ligand-receptor interactions with epithelial cells acting as ligands **(E)** and receptors **(F)** in the PCa group.

## 4 Discussion

PCa is a high-incidence malignancy in men and a leading cause of male cancer-related deaths (29). The influence of environmental pollutants on the development of PCa has attracted considerable attention in recent years. Novel environmental risk factors such as microplastics and their additives have been detected in human prostate tissues; however, the causal relationship and molecular mechanisms linking them to PCa remain poorly understood (7, 30, 31). The present *in vitro* experiments first demonstrated that ATBC significantly enhanced the proliferation, migration, and invasion of LNCaP cells. By integrating network toxicology, single-cell transcriptomics, Mendelian randomization, and multiple machine learning algorithms, this study further revealed that ATBC may drive the malignant progression of PCa by modulating key genes to interfere with metabolic reprogramming, cell migration, and invasion.

Network toxicology analysis of the potential target genes of ATBC revealed that their functions were also enriched in pathways closely associated with tumor metastasis, such as the regulation of vascular endothelial cell migration, membrane rafts, and Toll-like receptor binding. This finding is consistent with the *in vitro* results of the present study and with previous reports demonstrating that microplastics can promote tumor cell metastasis (13, 32).

Based on multidimensional analyses, two key genes were identified. LPCAT1 is a key enzyme in the phosphatidylcholine metabolic pathway that catalyzes the conversion of lysophosphatidylcholine to phosphatidylcholine; aberrant changes in phosphatidylcholine levels are considered a metabolic hallmark of tumorigenesis and tumor development (33). In this study, LPCAT1 was found to be markedly upregulated in PCa tissues, and the protein-level high expression was further confirmed by immunohistochemistry from the HPA database. LPCAT1 was significantly correlated with monocytes and immature dendritic cells, suggesting its potential involvement in PCa progression through remodeling of the tumor immune microenvironment (34). Moreover, GSEA enrichment analysis indicated that LPCAT1 exhibited a notable upregulation in pathways such as regulation of the actin cytoskeleton and ECM-receptor interaction. Combined with the virtual knockout results, these observations imply that LPCAT1 may influence PCa progression via pathways highly relevant to cell migration and invasion. This aligns with previous findings that silencing LPCAT1 reduces the proliferation and invasion abilities of PCa cells (35). It has been reported that elevated LPCAT1 expression in PCa tissues can serve as a prognostic biomarker for clinical outcomes after prostatectomy (36). Coupled with the present molecular docking result showing that ATBC can tightly bind to the LPCAT1-encoded protein, it is plausible that ATBC abnormally activates LPCAT1, remodels phospholipid metabolism, and upregulates cytoskeleton regulatory pathways, thereby enhancing the migratory and invasive capacities of PCa cells.

PDK1 is a negative regulatory kinase of the pyruvate dehydrogenase complex that forces cellular metabolism toward aerobic glycolysis, which is particularly crucial in tumors (37). The present GSEA analysis revealed that PDK1 was enriched in the calcium signaling pathway and significantly downregulated the oxidative phosphorylation pathway, fitting well with the role of PDK1 as a metabolic switch. Previous studies have indicated that PDK1 can function by regulating calcium ion infiltration into mast cells, which is consistent with the results of the GSEA and immune microenvironment analyses in this study, suggesting that PDK1 may influence the tumor microenvironment of PCa through mast cells (38). In addition, PDK1-promoted glycolysis leads to lactate accumulation and microenvironment acidification, thereby aggravating the invasive behavior of malignant cells (39). Prior functional studies have likewise shown that PDK1 knockdown significantly reduces the migration and invasion of PCa cells (40). Together with the molecular docking finding that ATBC binds tightly to the PDK1-encoded protein, these results suggest that ATBC may aberrantly activate PDK1, further strengthening the metabolic adaptability of tumor cells and providing energy and material support for their migration and invasion under high metabolic stress. Additionally, this study found significantly upregulated PDK1 mRNA expression in the transcriptomic cohort, though HPA immunohistochemical data showed no obvious rise in protein staining. This discrepancy may stem from post-transcriptional regulation, protein stability, small HPA sample size or tissue-specific translational regulation (41). Thus, PDK1 protein-level validation in clinical cohorts is required before medical translation.

Through single-cell sequencing analysis, the present study further identified epithelial cells as the key cell type in PCa at the cellular level. Among the epithelial cell subclusters, Double-Negative Basal-like Epithelial Cells exhibited the highest TGF-β signaling pathway activity. In advanced tumors, TGF-β signaling often shifts from a tumor suppressor to a tumor promoter, enhancing the migration and metastasis of epithelial cancer cells by inducing epithelial-mesenchymal transition (42). Pseudotime analysis demonstrated that the expression levels of LPCAT1 and PDK1 gradually increased during the mid-to-late stages of the cell differentiation trajectory, suggesting that these two genes may be closely associated with the differentiation and maturation of epithelial cells as well as metabolic adaptation during malignant transformation (43, 44). Cell communication analysis revealed a more complex intercellular communication landscape in the PCa microenvironment, involving vascular remodeling, inflammatory responses, and migration and invasion. These findings imply that LPCAT1 and PDK1, as targets of ATBC, may not only act within tumor cells but also synergistically promote tumor progression by influencing the tumor microenvironment through their potential association with epithelial cells.

Although this study used virtual knockout, molecular docking and MR analysis to explore the functions and potential regulatory mechanisms of ATBC target genes, there are still several limitations relying on public databases and experimental verification at the cellular level, which to some extent affects the extrapolability and clinical translation of the results of this study. Future studies should further explore the detailed regulatory mechanism of ATBC. Surface plasmon resonance (SPR), isothermal titration calorimetry (ITC) or cellular thermal shift assay (CETSA) can be used to verify the binding between ATBC and LPCAT1/PDK1. *In vivo* experiments such as constructing knockout models via siRNA/CRISPR technology or establishing LNCaP xenograft models in immunodeficient mice exposed to ATBC can be carried out to evaluate the expression of LPCAT1/PDK1. Clinical prostate tissue samples can be collected for immunohistochemistry (IHC) assays to verify the protein expression of LPCAT1 and PDK1. More datasets can be incorporated into the analysis, such as prostate tissue eQTL, multi-ethnic cohorts and clinical data, to improve extrapolability. Environmental ATBC exposure levels can be measured to build a physiologically based pharmacokinetic (PBPK) model, which will be compared with human biomonitoring data to clarify whether the pro-migratory effect exists within environmentally relevant exposure ranges, and further assess the biological effects of ATBC associated with human exposure.

## 5 Conclusion

This study combined *in vitro* cell experiments and multi-level bioinformatic analysis to reveal the potential mechanism by which the microplastic-associated compound ATBC and its toxic targets (LPCAT1 and PDK1) promote the migration and invasion of prostate cancer (PCa). Among them, LPCAT1 may remodel phosphatidylcholine metabolism, enhance actin cytoskeleton and extracellular matrix-receptor interactions, thereby facilitating cytoskeleton rearrangement and cell-matrix adhesion. PDK1 may induce glycolytic shift, inhibit oxidative phosphorylation and regulate calcium signaling pathways, thereby altering immune infiltration status, supporting tumor invasion and constructing a favorable microenvironment. In summary, ATBC exposure may potentially promote PCa metastasis through aberrant activation of LPCAT1 and PDK1. This study not only explores the potential effects of LPCAT1 and PDK1 on the malignant behaviors of PCa, but also provides novel potential biomarkers as well as prospective intervention targets for evaluating metastasis risks associated with exposure to microplastic environmental pollutants.

## Data Availability

The datasets (GSE46602, GSE38241, GSE316750, GSE181294, GSE193337, GSE176031, GSE153892, GSE221603, and GSE185344) supporting the conclusions of this article are available in the [GEO] repository, [https://www.ncbi.nlm.nih.gov/geo/]. The dataset (ebi-a-GCST90018905) supporting the conclusions of this article is available in the [IEU Open GWAS] repository, [https://gwas.mrcieu.ac.uk/]. The raw data supporting the conclusions of this study are included within the article and its **Supplementary Materials**. Further inquiries regarding the data can be directed to the corresponding authors.

## Supplementary material

Supplementary Figure 1 Single-cell data preprocessing and principal component analysis (PCA) analysis. (**A–F**) Distribution plots of nFeature_RNA, nCount_RNA, and mitochondrial gene proportion before and after quality control in datasets GSE181294 (A), GSE193337 **(B)**, GSE176031 **(C)**, GSE153892 **(D)**, GSE221603 **(E)**, and GSE185344 **(F)**. **(G)** Visualization charts of PCA before and after elimination of batch effects. **(H)** PCA chart after removing double-cell data points.

Supplementary Figure 2 Screening of highly variable genes and principal component analysis (PCA). **(A)** Screening of highly variable genes, with highly variable genes highlighted in yellow. **(B)** Cell clustering linear dimensionality plot. **(C)** Combined plot for principal component dimension screening, including 1. scree plot; 2. cumulative variance explained plot; 3. marginal gain plot of individual PCs; 4. second derivative curvature plot.

Supplementary Figure 3 Data processing workflow for epithelial cells subpopulation clustering. **(A, B)** Visualization charts of principal component analysis (PCA) before and after batch effect removal; before removal **(A)**, after removal **(B)**. **(C)** PCA linear dimension plot. **(D)** Comprehensive plot for principal component dimension screening, including: 1. Scree plot; 2. Cumulative explained variance plot; 3. Marginal gain plot of each principal component; 4. Second-order derivative curvature plot.

Supplementary Figure 4 Basic Toxicity Model Report of ATBC.

Supplementary Figure 5 MR analysis of PDK1 and NEU1. **(A–D)** MR scatter plot **(A)**, forest plot **(B)**, funnel plot **(C)**, and leave-one-out plot **(D)** of PDK1 and NEU1 gene.

Supplementary Figure 6 Ligand-receptor interaction analysis in the prostate cancer (PCa) group and control group. **(A–B)** Bubble plot of ligand-receptor interaction pairs between different cell types in the PCa group **(A)** and control group **(B)**.

Supplementary Table 1 Quality control of single-cell data.

Supplementary Table 2 Quantitative evaluation results of Batch effects.

Supplementary Table 3 Number of cells after single-cell quality control (QC) for each dataset.

Supplementary Table 4 List of all DEGs.

Supplementary Table 5 ATBC toxicity target genes.

Supplementary Table 6 GO enrichment results of candidate genes.

Supplementary Table 7 KEGG enrichment results of candidate genes.

Supplementary Table 8 F-statistic corresponding to the instrumental variable of LPCAT1.

Supplementary Table 9 F-statistic corresponding to the instrumental variable of PDK1.

Supplementary Table 10 F-statistic corresponding to the instrumental variable of NEU1.

Supplementary Table 11 GSEA enrichment of LPCAT1.

Supplementary Table 12 GSEA enrichment of PDK1.

Supplementary Table 13 Correlation analysis between key genes and immune cell infiltration.

Supplementary Table 14 GO enrichment results from gene knockdown analysis.

Supplementary Table 15 KEGG enrichment results from gene knockdown analysis.
